# Characterization of the first double-stranded RNA bacteriophage infecting *Pseudomonas aeruginosa*

**DOI:** 10.1038/srep38795

**Published:** 2016-12-09

**Authors:** Yuhui Yang, Shuguang Lu, Wei Shen, Xia Zhao, Mengyu Shen, Yinling Tan, Gang Li, Ming Li, Jing Wang, Fuquan Hu, Shuai Le

**Affiliations:** 1 Department of Microbiology, Third Military Medical University, Chongqing, 400038, China

## Abstract

Bacteriophages (phages) are widely distributed in the biosphere and play a key role in modulating microbial ecology in the soil, ocean, and humans. Although the role of DNA bacteriophages is well described, the biology of RNA bacteriophages is poorly understood. More than 1900 phage genomes are currently deposited in NCBI, but only 6 dsRNA bacteriophages and 12 ssRNA bacteriophages genome sequences are reported. The 6 dsRNA bacteriophages were isolated from legume samples or lakes with *Pseudomonas syringae* as the host. Here, we report the first *Pseudomonas aeruginosa* phage phiYY with a three-segmented dsRNA genome. phiYY was isolated from hospital sewage in China with the clinical *P. aeruginosa* strain, PAO38, as a host. Moreover, the dsRNA phage phiYY has a broad host range, which infects 99 out of 233 clinical *P. aeruginosa* strains isolated from four provinces in China. This work presented a detailed characterization of the dsRNA bacteriophage infecting *P. aeruginosa.*

Bacteriophages (phages) are estimated to be the most abundant organisms on earth, with key roles in shaping the composition of microbial ecology by lysing bacteria or transferring genes^1^,^2^. Despite the numerous available studies on DNA bacteriophages, the biology of RNA bacteriophages has been poorly investigated. Recently, Siddharth *et al*. identified the partial genome sequences of 122 RNA bacteriophage phylotypes by metagenomic sequencing^2^. Their results indicated that RNA phages are globally distributed in numerous microbial communities. More than 1900 phage genomes are currently deposited in NCBI. However, only the genome sequences of 6 double-stranded RNA (dsRNA) bacteriophages (phi6, phi8, phi12, phi13, phi2954, and phiNN) and 12 single-stranded RNA (ssRNA) bacteriophages are available in the GenBank Genomes database as of 1 September, 2016 (Supplementary Tables S1 and S2). Therefore, more unrecognized RNA bacteriophages have yet to be discovered.

The discovery of dsRNA phages dates back to 1973, when Vidaver *et al*. identified a lipid-containing bacteriophage, phi6, with a three-segmented dsRNA genome^3^. The characterization of these viruses has improved the understanding of dsRNA virus biology, such as its replication, virus assembly, genome packaging, and phage–host interactions^4^,^5^,^6^,^7^,^8^,^9^. All dsRNA phages are classified in the Cystoviridae family; phi6 is a representative member of Cystoviridae. The three genome segments of phi6 are designated L (6374 bp), M (4063 bp), and S (2948 bp). Currently, more than 10 dsRNA phages have been isolated; 6 of which (phi6, phi8, phi12, phi13, phi2954, and phiNN) have genome sequences deposited in GenBank^10^,^11^,^12^,^13^,^14^,^15^.

We previously isolated several dsDNA phages infecting *Pseudomonas aeruginosa*^16^,^17^. Here, we report a dsRNA phage phiYY isolated from the sewage of Southwest Hospital, with the clinical *P. aeruginosa* strain, PAO38, as the host. Compared with the previously identified dsRNA phages from legume samples or lakes, phiYY was isolated from hospital sewage and was the first dsRNA phage known to infect the opportunistic pathogen *P. aeruginosa*.

## Results and Discussion

### Biological characteristics of phiYY

**Figure 1:**
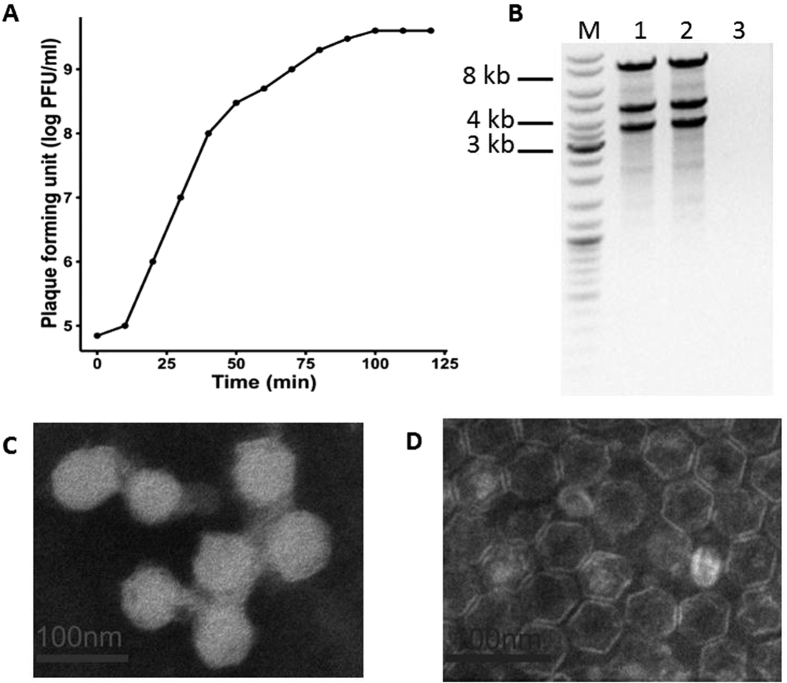
Biological characteristics of the phage phiYY. (**A**) One-step growth curve of phiYY. (**B**) Agarose gel electrophoresis of genomic segments. M: dsDNA marker, 1: phiYY genome, 2: phiYY genome digested with DNase, and 3: phiYY genome digested with RNase. (**C**) TEM image of phage particles. (**D**) TEM image of chloroform-treated phage particles. The scale bar indicates 100 nm.

Bacteriophage phiYY was isolated from sewage of Southwest Hospital, Chongqing, China, with *P. aeruginosa* PAO38 as the host. phiYY forms clear plaques (2–4 mm in diameter) on double agar plates. Phage particles were very sensitive to chloroform and could not form any plaques after chloroform treatment. The chloroform sensitivity of phiYY indicates a lipid membrane. The optimum multiplicity of infectivity (MOI) of phiYY was observed as 0.1 (phage:host = 1:10). The one-step growth curve of phiYY showed that its latent period is approximately 10 min and that its burst period is approximately 50 min (Fig. 1A), which is considerably faster than that of phi6. However, the average burst size (10–14) was much lower than that of phi6 (125–400). Enzyme digestion showed that phiYY contained three double-stranded RNA segments, which can be digested by RNase but not DNase (Fig. 1B). Transmission electron microscopy (TEM) revealed that the mature particle was tailless, spherical, and approximately 50 nm in diameter (Fig. 1C). However, the chloroform-treated particles were icosahedral symmetric structures (Fig. 1D).

### Identification and organization of phiYY genes

The dsRNA genome of phiYY was reverse transcribed into complementary DNA (cDNA) fragments and sequenced by next-generation sequencing. The sizes of the segments were 3,004 (S), 3,862 (M), and 6,648 (L) bp, similar to the genome segments of phi6, which are 2948 (S), 4063 (M), and 6374 (L) bp. The GC-content was 57.46%, 58.91%, and 59.34% for phiYY S, M, and L, respectively.

**Figure 2:**
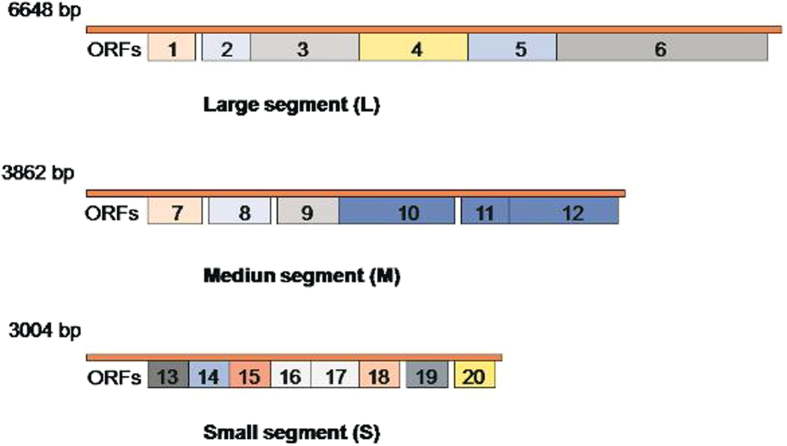
Genomic maps of the phage phiYY. Twenty potential open reading frames (ORFs) were predicted in the phiYY genome.

A total of 20 potential open reading frames (ORFs) were predicted from the phiYY genome. (Fig. 2). Table 1 lists the putative functions of each gene. Among the 20 predicted proteins of phiYY, 70% have putative functions. The majority of the phiYY proteins are structural proteins, such as the procapsid protein, host attachment protein, nucleocapsid shell protein, muramidase, and RNA-dependent RNA polymerase (RdRP). Like most RNA viruses, phiYY likely packed RdRP into the virus during the infection cycle for later use to replicate phage RNA upon host infection.

Protein BLAST revealed that most structural proteins of phiYY are similar to those of other dsRNA bacteriophages. The *orf3* gene, which encodes a polymerase^4^,^18^, showed 71% similarity to RdRP of phi13. The Orf5 protein was predicted to be an NTPase with 35% and 47% similarity to phi6 and phi13, respectively. NTPase is required for genomic packaging in phi6^19^. We also identified a muramidase, which showed 45% similarity to phi13, from segment S. Muramidase is a phage-encoded lysin that damages bacterial cell walls to release phage particles during the last step of the phage infection cycle^20^,^21^. Given the emergence of multi-drug resistant bacteria, phage lysin has been suggested to be a potential antimicrobial agent^22^. However, all the currently tested lysins were cloned from dsDNA phages. Thus, the efficiency of dsRNA phage-encoded muramidase against *P. aeruginosa* should be tested in the near future.

Only 6 putative proteins were predicted with unknown functions. By contrast, most dsDNA phage genome contains numerous genes with unknown functions^17^,^23^; some potentially encode virulent genes. Potential virulence is a safety concern for dsDNA phage therapy. However, dsRNA phage genomes are very concise and hypothetical proteins are very limited. Therefore, phiYY is less likely to carry a toxin gene. This feature might be advantageous for dsRNA phage-based phage therapy.

### Identification of phiYY structural proteins

**Figure 3:**
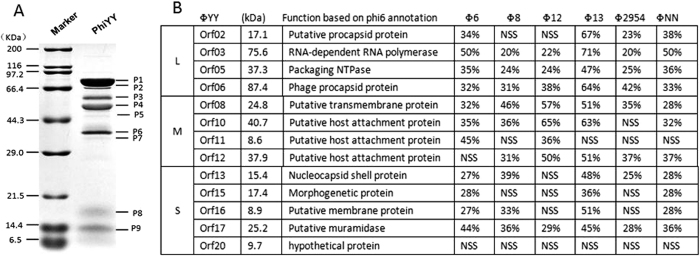
Identification of phiYY structural proteins. (**A**) SDS-PAGE analysis of phage structural proteins visualized in 12% (w/v) gel. (**B**) Structural proteins detected by HPLC-MS and their similarity to other dsRNA phages.

Structural proteins of phiYY were analyzed by sodium dodecyl sulfate-polyacrylamide gel electrophoresis (SDS-PAGE). The migration of the proteins was similar to that of other Cystoviridae (Fig. 3A). A total of 9 proteins were detected with molecular weights of 10–80 kDa.

To detect all the structural proteins, we performed SDS-PAGE for 30 min. We performed HPLC-MS analysis with the excised band containing the mixture of all the structural proteins (Fig. 3B). All the structural proteins were detected. Interestingly, we also identified a hypothetical structural protein, Orf20, without similarity to other phages. It is an additional structural protein absent in other dsRNA phages. Given that protein function cannot be determined by BLAST, further investigations are necessary to characterize the function of Orf20.

### Phylogenetic analysis

**Figure 4:**
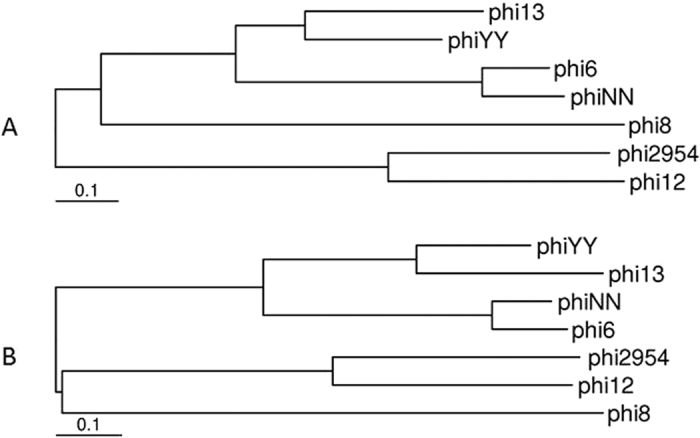
Phylogenetic trees showing the relationships between phiYY and other dsRNA phages based on nucleotide sequence comparisons of the major capsid protein (**A**) and L segment (**B**).

Phylogenetic trees were generated by MEGA6 based on the nucleotide sequence alignment of Orf06 with the major capsid protein from other dsRNA phages, phi6, phi8, phi12, phi13, phi2954, and phiNN (Fig. 4A). The nucleotide sequences of the phiYY L segment were also compared (Fig. 4B). The two generated phylogenetic trees indicated that phiYY and phi13 are closely related. The *Pseudomonas* phage phi13 was isolated from the leaves of the radish plant with *P. syringae* as the host strain^15^.

### Host range

**Figure 5:**
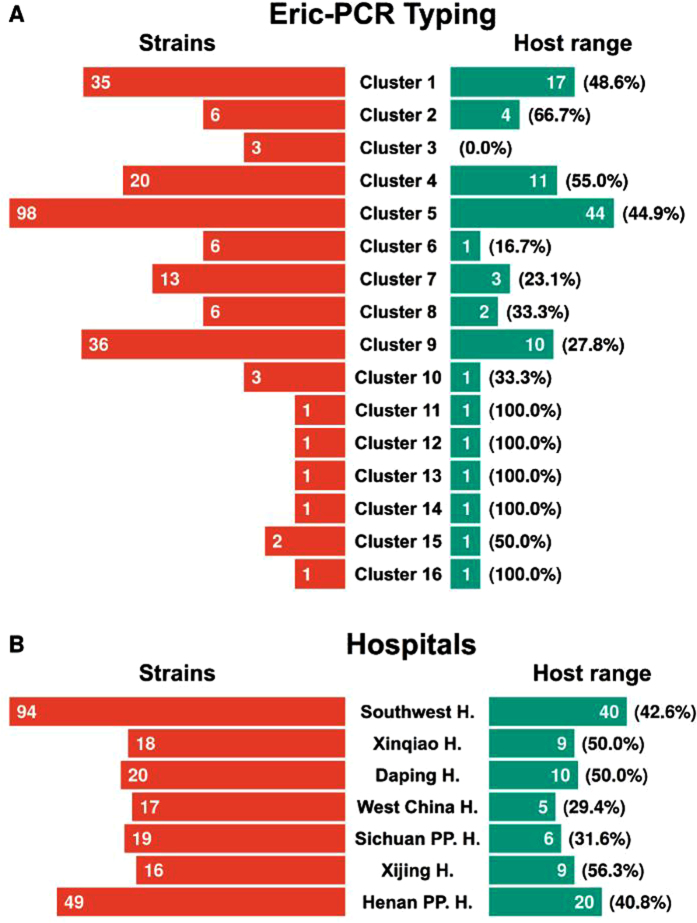
(**A**) ERIC-PCR typing of the collected *P. aeruginosa* strains and the phage sensitivity rate for each cluster. (**B**) *P. aeruginosa* strains collected from each hospital and the phage host range for clinic isolates from each hospital.

To characterize the host range of phiYY, we tested the phage against a panel of 233 isolated clinical strains and the type strain PAO1. The clinical strains were collected from seven hospitals from four provinces, as described in the Materials and Methods section. The number of clinical strains from each hospital is indicated in Fig. 5B.

To test the genetic diversity of our collected strains, we performed *Enterobacteriaceae* repetitive intergenic consensus polymerase chain reaction (ERIC-PCR)^24^. Supplementary Fig. S1 is a representative image of the ERIC-PCR profiles from *P. aeruginosa* strains. A total of 16 clusters were generated for the analyzed isolates, which were designated as Clusters 1 to 16. Figure 5A shows that the majority of our collections can be assigned to Cluster 5 (98), Cluster 9 (36), Cluster 1 (35), and Cluster 4 (20). According to the ERIC-PCR study, our collected isolates exhibit high genetic diversity, which can be used to assay the host range of phiYY.

We used the dot plaque assay to determine the resistance/susceptibility of all the strains against phiYY. The formation of a clear plaque in the bacterial lawn indicated susceptibility. Dot plaque assays were repeated to validate the results. In total, 233 clinical strains and PAO1 were tested. The host range was 42.3% (99 out of 234). Supplementary Table 2 shows the detailed resistance or susceptibility map.

phiYY was isolated from Southwest Hospital; hence, we collected 94 clinical strains from the same hospital. The collected strains were classified into 11 different clusters by ERIC-PCR (Supplementary Table S3). 42.6% of these strains are sensitive to phiYY. By contrast, the majority of the bacterial isolates from two hospitals in Sichuan Province were not lysed by phiYY (Fig. 5B). Additionally, phiYY did not infect PAO1. Overall, our *P. aeruginosa* collections are genetically diverse and phiYY infects 42.3% of these strains.

*P. aeruginosa* is an important Gram-negative opportunistic pathogen that causes serious infections in cystic fibrosis patients, ICU patients, and other immune-compromised individuals^25^,^26^. Given that *P. aeruginosa* is becoming resistant to most antibiotics, a common infection may be fatal. Therefore, the feasibility of treating bacterial infections with bacteriophages has been extensively studied^27^,^28^,^29^,^30^,^31^. Several studies have reported the efficacy of phage therapy against *P. aeruginosa*. The Phagoburn project was recently launched to systemically evaluate the safety and efficacy of phage therapy against *P. aeruginosa* and *E. coli* in burn patients^32^. Interestingly, all the phages used in these studies are DNA phages. The effectiveness of dsRNA phage therapy has yet to be reported.

dsRNA phages are possible alternative choices for phage therapy. First, the dsRNA phage phiYY has a broad host range and can be used alone or as a component of a phage cocktail. Second, dsRNA phages have a higher mutation rate and host range mutation rate^33^,^34^. The host range mutation frequency of the dsRNA bacteriophage phi6 is approximately 3 × 10^−4^. Therefore, the high host range mutation rate of dsRNA phage can be used to expand or change the phage host range to better fit phage therapy.

## Conclusions

This work presented a detailed analysis of the first dsRNA bacteriophage infecting *P. aeruginosa*. Our work enhances the current understanding of RNA phage biology and provides an alternative choice for phage therapy. Given that phiYY infects 99 out of 233 clinical *P. aeruginosa* strains, we are interested in further expanding the dsRNA phage host range by evolution experiments and genetic engineering. We will also test the effectiveness of dsRNA phage therapy as compared with dsDNA bacteriophages in animal models.

## Materials and Methods

### Bacteria and bacteriophage growth conditions

The *P. aeruginosa* PAO1 strain was maintained in our lab. A total of 233 clinical strains was collected from the Southwest Hospital (Chongqing, China), Daping Hospital (Chongqing, China), Xinqiao Hospital (Chongqing, China), West China Hospital (Sichuan, China), Sichuan Provincial People’s Hospital (Sichuan, China), Xijin Hospital (Xi’an, China), and the Henan Provincial People’s Hospital (Henan, China). All of the bacteria were grown at 37 °C in LB medium. phiYY was isolated from Southwest hospital sewage as previously described^27^.

### Transmission electron microscopy

The morphology of the purified phage and the chloroform-treated phiYY was observed by transmission electron microscopy as previously described^25^. Chloroform treatment was performed by vigorously shaking a mixture of 1 ml phiYY and 1 ml chloroform for 2 min. The mixture was centrifuged at 13,000 × *g* for 1 min. The phage particles were collected from the uppermost layer.

### One-step growth curve

To determine the one-step growth curve of phiYY, we used the methods described by Lu *et al*.^22^.

### Bacteriophage RNA isolation

phiYY virions were purified as previously described. Briefly, phiYY was inoculated into the log phase host in LB medium and cultured with aeration at 37 °C for 6 h. The phage lysate was centrifuged for 10 min at 10,000 × *g* and passed through a 0.22-μm filter. The phages were further purified by PEG8000 precipitation.

The phage dsRNA genome was isolated from the purified phage particles with the Tri Reagent extraction kit (Sigma-Aldrich). RNA integrity and size distribution were assessed on a 1% (w/v) agarose gel and visualized with ethidium bromide.

### Bacteriophage genome sequencing and bioinformatics analysis

Phage dsRNA genome was reverse-transcribed into cDNA with random oligo primers (SuperScript^®^ III First-Strand Synthesis System for RT-PCR, Invitrogen). Then, cDNA was sequenced by the semiconductor sequencer Ion Torrent Personal Genome Machine (PGM, ThermoFisher). The data generated from the genomic library was approximately 12 M with an average read length of 223 bases. The read data was assembled by the de novo assembly algorithm Newbler Version2.9 with default parameters.

phiYY genes were predicted with RAST (http://rast.nmpdr.org/)^35^ and fgenesV (http://linux1.softberry.com/berry.phtml?topic=virus&group=programs&subgroup=gfi ndv). The results were merged manually. DNA and protein sequences were scanned for homologs with BLAST^36^.

### Proteomic analysis of phage structural proteins

Proteomic analysis was performed as previously described^23^. Briefly, purified particles were denatured with heat and loaded onto a 15% (w/v) polyacrylamide gel. Proteins were stained with Coomassie Brilliant Blue R250 dye and washed with methanol–acetic acid–H2O.

To identify phage structural proteins, we performed the above mentioned experiment for 30 min. The protein band that included all the structural proteins was excised from the gel for HPLC-MS analysis. HPLC-MS data were processed by the Agilent Spectrum Mill proteomics software to allocate each protein to the corresponding gene.

### ERIC-PCR typing

Bacterial genomic DNA was extracted with TIANamp Genomic DNA Kit (DP304 Beijing, China). PCR amplification was performed with the following primers^24^. ERIC1 (5′-ATGTAAGCTCCTGGGGATTCAC-3′) and ERIC2 (5′-AAGTAAGTACTGGGGTGAGCG-3′). Each ERIC-PCR test was performed twice to ensure the conformity of each fingerprint.

The TIFF image was created from a photograph taken with the UV Gel Doc system (BIO-RAD, USA). The DNA banding patterns were entered into a database in ImageLab 2.0 software (BIO-RAD, USA) to automatically obtain and analyze the images. The ERIC-PCR patterns were interpreted and compared as previously described^24^. Similarity analysis was calculated with the Dice coefficient and the unweighted pair group average (UPGMA) for cluster analyses. When 80% or more similar bands were detected, these colonies were classified in the same cluster.

### Host range analysis by dot plaque assay

Phage sensitivity was determined by dot plaque assay. Briefly, 5 ml of molten 0.7% LB agar containing 100 μl of each test bacterial culture was overlaid on 1.5% LB agar plates. Subsequently, 1 μl of phiYY (~10^9^ PFU/ml) was spotted on the soft agar. Phage resistance/susceptibility was determined by the formation of clear plaques after overnight culture at 37 °C.

### Nucleotide sequence accession number

phiYY genomic sequence was submitted to GenBank. The accession numbers for segments L, M, and S are KX07420, KX074202, and KX074203, respectively.

## Additional Information

**How to cite this article**: Yang, Y. *et al*. Characterization of the first double-stranded RNA bacteriophage infecting *Pseudomonas aeruginosa. Sci. Rep.* **6**, 38795; doi: 10.1038/srep38795 (2016).

**Publisher's note:** Springer Nature remains neutral with regard to jurisdictional claims in published maps and institutional affiliations.

## Supplementary Material

Supplementary Information

## Figures and Tables

**Table 1:** List of ORFs for phiYY.

Segment	ORFs	Nucleotide position	Length (aa)	Mass (Da)	pI	Best match (aa identity)	E-value	Function
L	orf01	208–519	103	11593	5.76	NAD-glutamate dehydrogenase (29%)	9.2	hypothetical protein
	orf02	591–1070	159	17145	4.56	phi-13Lp1 (67%)	7e-54	putative procapsid protein
	orf03	1070–3100	676	75693	5.62	phi-13Lp2 (71%)	0.0	RNA-dependent RNA polymerase
	orf04	2953–5211	752	81201	5.93			hypothetical protein
	orf05	3097–4149	350	37295	6.47	phi-13Lp3 (47%)	6e-90	packaging NTPase
	orf06	4159–6576	805	87458	6.52	phi-13Lp4 (64%)	0.0	phage procapsid protein
M	orf07	245–694	149	16285	9.33			hypothetical protein
	orf08	749–1453	234	24843	10.18	phi-13Mp2 (57%)	5e-54	putative transmembrane protein
	orf09	1518–1955	145	16370	9.72	KIZ kizuna centrosomal protein (37%)	6.1	hypothetical protein
	orf10	1377–2489	370	40695	5.34	phi-12Mp3 (65%)	0.0	putative host attachment protein
	orf11	2499–2741	80	8582	4.37	phi-12Mp4(36%)	1e-04	putative host attachment protein
	orf12	2731–3810	359	37891	6.50	phi-13Mp5 (51%)	4e-116	putative host attachment protein
S	orf13	319–750	143	15412	6.37	phi-13Sp1 (48%)	2e-28	nucleocapsid shell protein
	orf14	707–1183	158	16159	9.44			hypothetical protein
	orf15	750–1244	164	17454	5.67	phi-13Sp2 (36%)	2e-28	morphogenetic protein
	orf16	1247–1495	82	8911	8.46	phi-13Sp3 (51%)	3e-05	putative membrane protein
	orf17	1497–2189	230	25174	9.23	phi-13Sp4 (45%)	4e-59	putative muramidase
	orf18	2156–2383	75	8419	10.63			hypothetical protein
	orf19	2553–2690	45	4859	11.62			hypothetical protein
	orf20	2724–2999	91	9712	10.18			hypothetical protein
