# Corrigendum: Characterization of the first double-stranded RNA bacteriophage infecting *Pseudomonas aeruginosa*

**DOI:** 10.1038/srep46853

**Published:** 2017-06-15

**Authors:** Yuhui Yang, Shuguang Lu, Wei Shen, Xia Zhao, Mengyu Shen, Yinling Tan, Gang Li, Ming Li, Jing Wang, Fuquan Hu, Shuai Le

Scientific Reports 6: Article number: 38795; 10.1038/srep38795 published online: 12 09 2016; updated: 06 15 2017.

This Article contains a typographical error in the Materials and Methods section, under subheading ‘Nucleotide sequence accession number’,

“The accession numbers for segments L, M, and S are KX07420, KX074202, and KX074203, respectively”.

should read:

“The accession numbers for segments L, M, and S are KX074201, KX074202, and KX074203, respectively”.

