# Supplementary material for: Characterization of the first double-stranded RNA bacteriophage infecting Pseudomonas aeruginosa
Source: Sci Rep. 2016 Dec 9;6:38795. doi: 10.1038/srep38795 (PMC5146939; doi:10.1038/srep38795)

Supplement information

Characterization of the first double-stranded RNA bacteriophage infecting *Pseudomonas aeruginosa*

Yuhui Yang [1], Shuguang Lu[1], Wei Shen [1], Zhao Xia[1], Mengyu Shen [1], Yinling Tan[1], Gang Li[1], Ming Li[1], Jing Wang[1], Fuquan Hu[1*], Shuai Le[1*]

1 Department of Microbiology, Third Military Medical University, Chongqing, 400038, China

* To whom correspondence should be addressed to: Fuquan Hu, Department of Microbiology, Third Military Medical University, Chongqing 400038, China. Tel.: 86-23-68752834. Fax: 86-25-68752834. E-mail: hufuquan2009@yahoo.cn; Shuai Le, Department of Microbiology, Third Military Medical University, Chongqing 400038, China.. E-mail: leshuai2004@qq.com;

Key words: *Pseudomonas aeruginosa*, double stranded RNA phage, Cystoviridae

Supplementary Table S1: List of all the ssRNA phages from GenBank Genomes database.

**Genome**	**Accession**	**Length**
**Acinetobacter phage AP205**	NC_002700	4268 nt
**Caulobacter phage phiCb5**	NC_019453	3762 nt
**Enterobacteria phage BZ13**	NC_001426	3466 nt
**Enterobacteria phage C-1 INW-2012**	NC_019920	3523 nt
**Enterobacteria phage FI**	NC_028902	4184 nt
**Enterobacteria phage FI sensu lato**	NC_004301	4276 nt
**Enterobacteria phage Hgal1**	NC_019922	3562 nt
**Enterobacteria phage M**	NC_019707	3405 nt
**Enterobacteria phage MS2**	NC_001417	3569 nt
**Enterobacteria phage Qbeta**	NC_001890	4215 nt
**Pseudomonas phage PP7**	NC_001628	3588 nt
**Pseudomonas phage PRR1**	NC_008294	3573 nt

Supplementary Table S2: List of all the dsRNA phages from GenBank Genomes database.

Genome	Isolated from	Accession	Length (nt)
**Pseudomonas phage phi 12**	U.S.A	NC_004174	2322
		NC_004175	4100
		NC_004173	6751
**Pseudomonas phage phi13**	U.S.A	NC_004170	2981
		NC_004171	4213
		NC_004172	6458
**Pseudomonas phage phi2954**	U.S.A	NC_012091	6501
		NC_012093	2578
		NC_012092	3606
**Pseudomonas phage phi6**	U.S.A	NC_003715	6374
		NC_003716	4063
		NC_003714	2948
**Pseudomonas phage phi8**	U.S.A	NC_003301	3192
		NC_003299	7051
		NC_003300	4741
**Pseudomonas phage phiNN**	Finnish	KJ957166	2945
		KJ957165	3814
		KJ957164	6503
**Pseudomonas phage phiYY (This study)**	China	KX074203	3004
		KX074202	3862
		KX074201	6648

Supplementary Table S3: The detailed phage sensitivity rate for each cluster and hospital. The number after "/" is the total strains in this cluster and the number before "/" is the number of phage sensitive colonies.

Hospital	Southwest Hospital	Xinqiao Hospital	Daping Hospital	West China Hospital	Sichuan Provincial People's Hospital	Xijing Hospital	Henan Provincial People's Hospital
**Cluster 1**	9/18	0/1	1/2			0/1	7/13
**Cluster 2**	0/2						4/4
**Cluster 3**	0/3						
**Cluster 4**	3/10	3/3	1/1	1/2	1/1	1/1	1/2
**Cluster 5**	18/26	4/10	2/2	4/11	5/16	6/11	5/22
**Cluster 6**	1/6						
**Cluster 7**	3/10			0/3			
**Cluster 8**			1/1				1/5
**Cluster 9**	4/15	0/1	5/14	0/1	0/2	1/2	0/1
**Cluster 10**	0/2						1/1
**Cluster 11**	1/1						
**Cluster 12**						1/1	
**Cluster 13**	1/1						
**Cluster 14**							1/1
**Cluster 15**		1/2					
**Cluster 16**		1/1					

Supplementary Fig. S1: A representative picture of the ERIC-PCR profiles from *P. aeruginosa* strains. M: DNA maker; lanes 1–16, *P. aeruginosa* isolates.

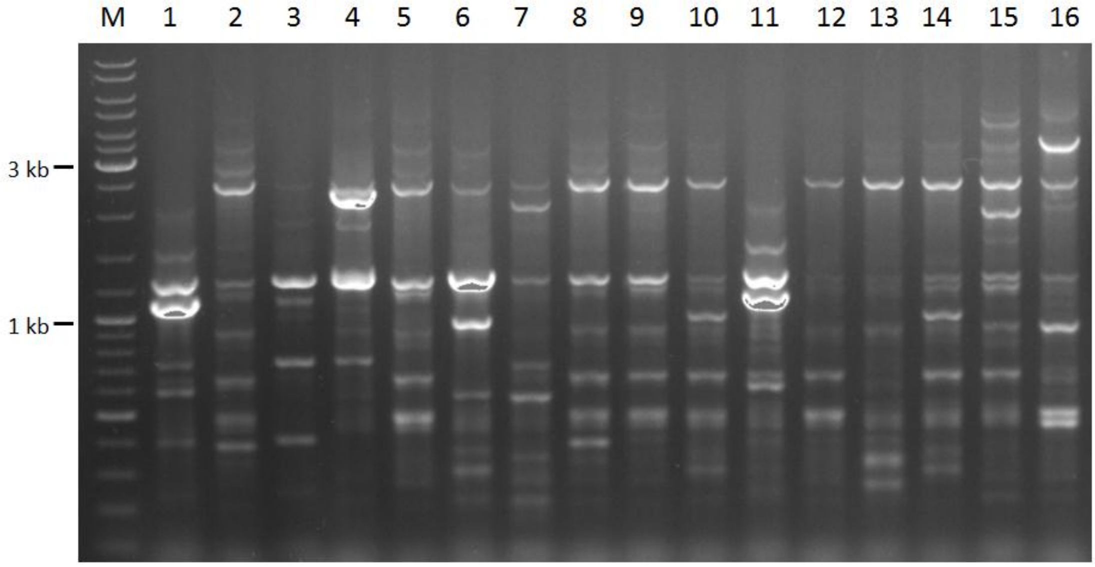

Supplement: Supplementary Information [file srep38795-s1.pdf]
